# Posterior Paralysis in Mules, from Entozoa in the Kidneys

**Published:** 1881-04

**Authors:** G. M. Bowler

**Affiliations:** Cincinnati


### ART. XI.—POSTERIOR PARALYSIS IN MULES, FROM ENTOZOA IN THE KIDNEYS.

THE following case of paralysis of the hind extremities of a large number of mules, arising from entozoa within the pelvis of the kidney, may prove of interest to some of your readers. The reason I have not published it before will be apparent to the reader as he proceeds.

A stock-raiser, residing about fifty miles from Cincinnati, called upon me some time ago in reference to a disease that was carrying off his mules in a very rapid manner.

His stock of mules, at the time he called upon me, amounted to about forty head, of two or three years old, and previous to this he had lost about nineteen or twenty.

He informed me that the mules which had died were affected in a very peculiar manner, and that all presented the same symptoms. The first noticeable symptom was an unsteady gait when walking, which gradually increased, until, when the patient lay down, he was unable to rise again, having lost the entire power of the hinder extremities, from the fact of their having become paralyzed.

Notwithstanding all this, their appetite appeared as good as when in perfect health. The owner informed me that their principal food was corn fodder, and as they lay on the ground appeared to enjoy their food immensely, although unable to walk. He furthermore informed me that some time ago a difficulty had arisen between himself and the owner of the farm adjoining his, in consequence of which they were at swords' points with each other, and in consequence of his neighbor also having a large number of mules, none of which had displayed any of the fatal symptoms; and taking into consideration the feud which had been existing between themselves for so long, it very naturally ocurred to the owner of the diseased animals that his neighbor was the sole cause of his misfortune, and that he had resorted to poison in order to be revenged on his enemy.

He at once consulted two able lawyers, stating the case in full; and so convinced were they of the guilt of the neighbor, that they at once entered suit, placing the damages at ten thousand dollars. After suit had been entered, it occurred to the legal gentlemen that a post-mortem examination would be next in order, and as there was no competent veterinarian in the neighborhood, two gentlemen of the medical profession were employed to make the autopsy. The conclusion from their examinations was, that the mules had been poisoned. The counsel for plaintiff was not, however, satisfied, and thought it would be better to have an examination by a veterinary surgeon, in consequence of which the owner called upon me, and requested that I would come to the farm and make another examination. On the following day I boarded the train and arrived at the farm at about seven P.M., too late, however, for business, so I concluded to postpone the examination until the following morning.

When morning came we proceeded to the barn where the mules were feeding, and the first thing I noticed was a number of the animals lying around the yard, feeding as though nothing was wrong with them; but on trying to make them rise, I found they were unable to use the hinder extremities.

I next selected the mule that appeared to me to be the worst affected, and with a blow from a hammer slaughtered him. I then carefully examined the various organs in order to discover the poisoning as described by the two physicians, but could find no such conditions present. After having examined the thoracic and abdominal viscera, I was about closing my exmination, when it occurred to me that one kidney was absent; that is, there was so little remaining as to constitute little more than what would form the pelvis of the organ that should have been present; in fact, nothing more than a fatty-looking substance about the size of a hen's egg. I carefully removed it, taking care to dissect out a portion of the ureter, which I laid aside. I then removed the fellow member, which presented a natural appearance as regards size, and this I also laid aside.

I next took the atrophied kidney, and by a stroke of the scalpel laid it open, when I discovered the pelvis swarming with entozoa, some of which were so minute that they had penetrated far into the ureter.

The fellow kidney was next examined, and found to be affected in like manner, but not to the same extent.

I then selected a second mule, the one which appeared to be the farthest advanced in the disease, and slaughtered him in the same manner as number one. In this case I found both kidneys considerably atrophied and containing a large number of the entozoa.

Mule number three was next slaughtered, being unable to rise; very similar conditions were presented in this case; each kidney, however, seemed to swarm with the entozoa, which were extremely small, presenting the appearance of a fine darning-needle.

I now concluded that my investigation had been pushed far enough to convince the greatest skeptic that the poisoning, as stated by the medical gentlemen, was purely imaginary. Here was positive evidence that the loss of power was entirely owing to the presence of a collection of parasites within the pelvis of the kidneys, producing atrophy of those organs, and resulting in paralysis of the hind extremities. The owner and several other gentlemen were perfectly satisfied as to the cause of the disease, and on the case being stated to his legal adviser, they at once withdrew the suite, without informing the defendant why or wherefore, and there it ended. At the request of the owner of the mules, I kept it out of print; the reason why must be obvious to every reader. I took the best specimen home with me, and on carefully examining the entozoa, found they belonged to the class known as the *Strongylus;* but never having met with a similar case, and wherein the conditions were so much alike in each subject, I thought it might possibly be of interest to some of your readers, who are liberal enough to acknowledge that there are many things yet to learn, even in the veterinary schools.

I would also state that I placed the balance of the mules under a course of treatment, which consisted of small doses of hydrochloric acid largely diluted with water, twice a day. A gradual improvement was observable from the time I commenced to use it, and the entire drove of mules was restored to apparent health.

CINCINNATI. Feb. 26, 1881. G. M. BOWLER, M.D.,V.S.

